# Effects of Dietary Fatty Acids on Lipid Traits in the Muscle and Perirenal Fat of Growing Rabbits Fed Mixed Diets

**DOI:** 10.3390/ani2010055

**Published:** 2012-02-22

**Authors:** Pier Giorgio Peiretti

**Affiliations:** Institute of Sciences of Food Production, National Research Council, via Leonardo da Vinci 44, 10095 Grugliasco, Italy; E-Mail: piergiorgio.peiretti@ispa.cnr.it; Tel.: +39-011-670-9233; Fax: +39-011-670-9297

**Keywords:** rabbit, fatty acids, n-3 PUFA, *longissimus dorsi* muscle, perirenal fat, oleic acid, linoleic acid, α-linolenic acid

## Abstract

**Simple Summary:**

Polyunsaturated fatty acids in human foods have been shown to have health benefits. We investigated the potential to incorporate them into rabbit meat by adding them to the diet. Good relationships between dietary fatty acids (FAs) and their content in *longissimus dorsi* muscle and perirenal fat of rabbits was established, especially the latter. The results should make it possible to enhance the polyunsaturated fatty acid content of rabbit meat, with benefits to the health of human consumers.

**Abstract:**

The aim of this study was to evaluate the effects of various raw materials (spirulina, curcuma, tomato pomace, false flax, linseed, chia, perilla seeds) as suitable polyunsaturated fatty acid n-3 (n-3 PUFA) sources, on the lipid traits in the *longissimus dorsi* muscle and perirenal fat of growing rabbits. The fatty acid (FA) analyses of the diets, carried out by gas chromatography, differed over a wide range on the basis of the highly varied ingredients in 27 experimental formulations. Among the 29 identified FAs, three from feeds were catabolized in the rabbits, five were *de novo* synthesized and stored chiefly in the muscle. It was possible to linearly characterize the incorporation from the feed to the muscle of 16 FAs. This study has confirmed that the dietary inclusion of various raw materials could be considered as a way of enriching the n-3 PUFA of rabbit meat. A proposal for the prediction of n-3 PUFA from dietary α-linolenic acid (C18:3 n-3) and a panel of another 10 FAs has been made for intramuscular fat (R^2^ = 0.94) and perirenal fat (R^2^ = 0.96).

## 1. Introduction

One of the main aims of meat researchers is to produce dietetic and healthy meat in order to reduce the saturated fatty acids (FAs) and increase the unsaturated FAs in fat deposits [1]. Rabbit meat has a good nutritional value and is highly valued because of its dietary properties, since it is a lean meat with a low-fat content and less saturated FAs and cholesterol than other meats [2]. These characteristics, together with the possibility of manipulating the composition of the FAs through diet, mean that rabbit meat could be valuable in human nutrition [3]. The data on the chemical composition of rabbit meat, especially those on the fat content and FA profile, exhibit a relatively pronounced diversity and depend on the feeding, age, genotype, breeding and/or physical activity of the animals as well as on the muscle type and the sex of the animals in question [4,5].

Rabbits, like other monogastric animals, are able to directly incorporate dietary FAs into adipose and intramuscular tissue lipids, thus making it possible to modify the FA profile of rabbits through the strategic use of unsaturated dietary fat sources [1]. Feeding has the highest impact on meat quality and different studies have shown that, after a month of treatment, the FA profile of rabbit muscles can effectively be modified when the rabbits are fed diets with different FA supplementations [6,7]. The administration of an enriched diet during the last two weeks of fattening is sufficient to increase the polyunsaturated fatty acids (n-3 PUFA) content of the meat, thus reducing costs in comparison to a longer treatment [8]. Muscle FA incorporation and reversibility have been demonstrated by Szabó *et al*. [9], who concluded that the dietary fat source can effectively modify the skeletal muscle FA profile when fed in a proper time interval.

FA levels vary a great deal on the basis of the nature of the rabbit diets [10]. The influence of the FA profile in the diet seems to be more pronounced on the FA composition of adipose tissue than intramuscular fat [10]. A great deal of research has been focused on increasing the n-3 PUFA content in rabbit meat through the diet. As for other monogastric animals, this increase may be performed by supplementing diets with vegetable oil or raw materials rich in n-3 PUFA content [11].

The effects of various raw materials (spirulina, curcuma, tomato pomace, false flax, linseed, chia, perilla seeds), as suitable unsaturated FA sources, have been the subject of eight experiments concerning the quality of rabbit meat [12,13,14,15,16,17,18,19]. Many of these studies have shown that supplementation is effective in improving the n-3 PUFA content, decreasing the n-6/n-3 ratio and reducing the saturation, atherogenic and thrombogenic indexes of the meat and fat, with consequent benefits on the nutritional quality of rabbit meat for consumers.

It was considered interesting to study the kinetics and quantitative relationship of FA deposits in rabbit meat. Therefore, the aim of the present research was to model the relationships between these FAs, and particularly in the rabbit tissues and feeds.

## 2. Experimental Section

The raw materials were tested according to the guidelines for applied nutrition experiments in rabbits [20]. The rabbits were housed individually under standard conditions at 22 °C ± 2 °C in wire cages at a height of 90 cm from the concrete floor. The type of diets, breed, initial mean weight and number of animals for each trial are reported in Table 1. All the diets were pelleted fresh and stored in darkness to avoid auto-oxidation of the lipid sources. The feeds and water were available *ad libitum*.

**Table 1:** Data set characteristics.

Reference	Dietsn ∑ 27	Raw material	Breed	Weight (g)	Animals n. ∑ 246
[12]	4	Spirulina / fat level^1^	New Zealand	2,805	16
[13]	3	False flax^2^	Crossbred	2,316	30
[14]	3	Chia^3^	Crossbred	1,433	30
[15]	3	Golden flaxseed^4^	Crossbred	2,074	30
[16]	4	Spirulina^5^	Crossbred	2,034	40
[17]	3	Perilla^6^	Crossbred	1,120	30
[18]	4	Curcuma / oils^7^	Crossbred	1,512	40
[19]	3	Tomato pomace^8^	Crossbred	1,166	30

^1^ Spirulina(*Spirulina platensis*) supplementation (0 and 1%) and fat level (3 and 13%). ^2^ False flax(*Camelina sativa *L.) seed supplementation (0, 10 and 15%). ^3^ Chia (*Salvia hispanica* L.) seed supplementation (0, 10 and 15%). ^4^ Golden flaxseed (*Linum usitatissimum* L.) supplementation (0, 8 and 16%). ^5^ Spirulina(*Spirulina platensis*) supplementation (0, 5, 10 and 15%). ^6^ Perilla (*Perilla frutescens*) seed supplementation (0, 5 and 10%). ^7^ Curcuma (*Curcuma longa*) supplementation (0 and 0.3%) and oils (with 4% palm oil and 4% mais oil. ^8^ Tomato (*Lycopersicon esculentum* Mill.) pomace supplementation (0, 3 and 6%).

The FA profile of the diets, as well as the perirenal fat and *longissimus dorsi* muscle of the rabbits have been reported in eight papers [12,13,14,15,16,17,18,19]. Lipid extraction was performed on the diets, meat, and fat samples according to Hara and Radin [21], while the transesterification of the FAs was carried out according to Christie [22], with the modifications described by Chouinard *et al*. [23]. The FAs were analyzed as their methyl esters. The analysis was carried out by gas chromatography, as reported by Peiretti *et al*. [14].

Linear regression models were developed from the individual data to study the changes in the measured FAs in the rabbit tissues. A complete model was formulated as:

FAijk = ai + biXjjk + Eijk

where i is the muscle (M) or the perirenal fat (P) tissue and b is the regression coefficient of the FA on X, which is the FA in the feed of the j^th^ group (1–27); k is the individual rabbit within the groups (1–246); E is the random error from the fitting. In this way it was possible to obtain information on the significance of the regression coefficient and on the rate of variation of the same FA in the two tissues (muscle and perirenal fat) through a contrast between the bM and bP coefficients.

The endogenous pathways of the FAs, were observed in some cases in the tissues but were absent in feed. Since many of these endogenous FAs are n-3 PUFA, a useful index that could be applied to dietetic rabbit meat is the total n-3 PUFA in the edible tissue. This variable, which was assessed in the muscle and in the perirenal fat, was related to all the FAs present in the feed through a regressive linear model as a possible precursor. Non significant variables were excluded from the final partial regression. In order to ascertain the overall representativeness of the diets in the tissues, on the basis of the relative amount of covariation between the diets and the FA profiles, three Pearson correlation coefficients were calculated on 3,792 diet-muscle-perirenal fat triplets concerning 16 available FAs, present in three stages.

**Table 2:** Statistics of the recovered or unrecovered FAs, over the whole range detected in the diets (n = 27), in the *longissimus dorsi* muscle (n = 246) and in the perirenal fat (n = 246).

FA	Diets			Muscle			Perirenal		
	Mean	SD	VC	Mean	SD	VC	Mean	SD	VC
C10:0	0	0	-	0	0	-	0.066	0.11	165%
C12:0	0.004	0.019	539%	0	0	-	0.12	0.13	110%
C14:0	0.43	0.66	155%	2.07	0.42	20%	1.87	0.43	23%
C14:1	0	0	-	0.063	0.12	194%	0.042	0.08	183%
C15:0	0	0	-	0.36	0.19	53%	0.48	0.10	20%
C16:0	15.81	7.16	45%	25.78	3.61	14%	23.39	4.34	19%
C16:1 n-7	0.040	0.085	211%	0.39	1.16	295%	0.68	1.26	185%
C16:1 n-9	0.24	0.25	104%	2.88	1.66	57%	1.70	1.21	71%
C16:3 n-4	0	0	-	0.04	0.12	280%	0	0	-
C17:0	0.56	1.00	180%	0.44	0.25	57%	0.42	0.25	60%
C17:1	0.008	0.032	381%	0.052	0.12	224%	0.10	0.15	154%
C18:0	3.43	1.48	43%	5.94	0.92	15%	6.48	2.32	36%
C18:1 n-7	0.45	0.42	93%	0.78	0.55	70%	0.62	0.49	79%
C18:1 n-9	22.81	7.25	32%	24.71	3.88	16%	23.85	5.56	23%
C18:2 n-6	36.43	13.66	37%	23.21	4.76	20%	27.00	6.57	24%
C18:3 n-3	13.15	13.67	104%	7.79	7.52	96%	9.98	10.39	104%
C18:3 n-6	0.10	0.33	341%	0.11	0.23	215%	0.14	0.28	205%
C18:4 n-3	1.97	5.37	273%	0	0	-	0	0	-
C20:0	0.25	0.23	91%	0	0	-	0.62	1.35	216%
C20:1 n-9	0.80	2.12	264%	0.33	1.03	317%	0.57	1.42	248%
C20:2 n-6	0.46	1.64	354%	0.060	0.19	315%	0.19	0.21	115%
C20:3 n-3	0	0	-	0.029	0.08	287%	0.025	0.06	248%
C20:3 n-6	0	0	-	0.24	0.72	294%	0	0	-
C20:4 n-6	0.052	0.12	230%	2.04	1.20	59%	0.074	0.17	228%
C20:5 n-3	0	0	-	0.023	0.08	341%	0	0	-
C21:0	0.21	0.63	302%	0.15	0.69	458%	0.034	0.07	191%
C22:1 n-9	0.18	0.66	375%	0	0	-	0	0	-
C22:4 n-6	0	0	-	0.15	0.28	185%	0	0	-
C22:6 n-3	0	0	-	0.084	0.24	286%	0	0	-
Others	2.64	3.19	121%	2.26	1.87	83%	1.60	0.99	62%
Raw avg.			*207%*			*173%*			*123%*

Blue: Catabolized FAs Red: *De novo* synthesis FAs

The data, expressed as part of the total FAs, were analyzed through regression and correlation analyses using the SPSS [24] software package (version 11.5.1 for Windows, SPSS Inc., USA).

## 3. Results and Discussion

Table 2 reports the mean content of the FAs that have been detected in the 27 diets and in the two rabbit tissues (246 samples of muscle and 246 samples of perirenal fat, respectively). The study has shown a very high variability of FAs in the diets that resulted from the variation coefficient (VC), which was only limited to less than 50% in 4 FAs but it exceeded 150% in 12 cases out of 20. The FA profiles of the diets, which differed according to the ingredients, are not analytically reported in the present study; as expected, the diets supplemented with oilseed (false flax [13], chia [14], linseed [15], and perilla [17]) had increased proportions of linoleic (C18:2 n-6) and α-linolenic acid (C18:3 n-3) with increasing supplementation level of seeds, while the unsupplemented diets or those supplemented with spirulina [12,16], curcuma [18], or tomato pomace [19] contained large amounts of palmitic (C16:0), oleic (C18:1 n-9) and linoleic acid.

Different groups of FAs can be observed in Table 2. Only two FAs were totally catabolized: stearidonic acid (C18:4 n-3), common in many diets, and erucic acid (C22:1 n-9), which was only present in the trial on false flax (*Camelina sativa* L.) seeds [13]. A lack of lauric acid (C12:0) and arachidic acid (C20:0) was observed in the muscle. Conversely five PUFA (C16:3 n-4, C20:3 n-6, C20:5 n-3, C22:4 n-6, and C22:6 n-3) were found in the muscle profile. Another three *de novo* synthesis FAs (C14:1, C15:0, and C20:3 n-3) were recovered in both tissues, while C10:0 was only found in the perirenal fat.

When considering at the VC in the muscle and in the perirenal profiles, six FAs (C14:0, C15:0, C16:0, C18:0, C18:1 n-9, and C18:2 n-6) were more stable (VC below 50%), four FAs (C16:1 n-9, C17:0, C18:1 n-7, and C18:3 n-3) showed slight variability with VC 50–100%, but the majority of FAs showed a very high degree of variability (VC > 150%). The raw average of the VC was 207, 173 and 123 % for the diets, the muscle and the perirenal fat, respectively.

**Table 3:** Linear regression equations of the fatty acids (FAs) of the *longissimus dorsi* muscle (M), the perirenal fat (P) and of the feed.

FA	R^2^	MSE	M	P	Sign.	bM	Sign.	bP	Sign.	b1#b2
C12:0	0.29	0.10	0.00	0.12	<.0001	0.00	1	0.33	0.2289	0.4163
C14:0	0.13	0.41	2.07	1.87	<.0001	0.17	<.0001	0.17	<.0001	0.9727
C16:0	0.44	3.13	25.80	23.37	<.0001	0.27	<.0001	0.37	<.0001	0.0071
C16:1 n-7	0.59	0.79	0.38	0.70	0.042	8.83	<.0001	12.41	<.0001	0.0000
C16:1 n-9	0.15	1.44	2.88	1.70	<.0001	-0.15	0.733	−1.08	0.0123	0.0000
C17:0	0.09	0.24	0.45	0.42	0.48	0.08	<.0001	0.06	0.0001	0.2995
C18:0	0.04	1.77	5.93	6.48	0.0001	0.22	0.010	−0.12	0.1513	0.0046
C18:1 n-7	0.88	2.61	0.76	0.64	0.22	1.30	<.0001	1.15	<.0001	0.0000
C18:1 n-9	0.71	0.18	24.65	23.94	<.0001	0.39	<.0001	0.66	<.0001	0.0000
C18:2 n-6	0.74	3.09	23.22	26.99	0.60	0.29	<.0001	0.41	<.0001	0.0000
C18:3 n-3	0.91	2.74	7.85	9.91	0.0083	0.50	<.0001	0.70	<.0001	0.0000
C18:3 n-6	0.84	0.10	0.10	0.14	0.028	0.61	<.0001	0.78	<.0001	0.0000
C20:0	0.27	0.87	0.00	0.62	<.0001	0.00	1	−2.54	<.0001	<.0001
C20:1 n-9	0.98	0.16	0.33	0.57	<.0001	0.47	<.0001	0.65	<.0001	0.0000
C20:2 n-6	0.13	0.20	0.06	0.19	<.0001	-0.01	0.1466	−0.03	<.0001	0.0295
C20:4 n-6	0.67	0.76	2.03	0.00	<.0001	4.04	<.0001	0.00	1	0.0000

Table 3 shows the tests of the different concentrations in the two tissues, and the regression coefficients of the FAs in the muscle and perirenal fat on the same FAs of the diets. In general, from this table it emerged that perirenal fat was more reactive than muscle in modifying its FA profile according to the diets and that the two coefficients where not significantly different in only 3 cases out of 16. The FA profile of the muscle, which is more homeostatic, reproduces that of the cell membrane much more than that of the adipocite, in which the characteristics of the depot fat are pre-eminent [25].

There was a lack of lauric acid and arachidic acid in the muscle, while arachidonic acid (C20:4 n-6) was incorporated in the muscle at a very high ratio (4.04). In the case of integration with a small quantity of erucic acid, which is sometimes present in oil by-products used for animal supplies, this was totally transformed and disappeared from the tissues. The myristic (C14:0), palmitic and oleic acids were more abundant in the muscle than in the perirenal fat; moreover, the myristic and palmitic acids were less influenced by diet than the oleic acid. The γ-linolenic acid (C18:3 n-6), C16:1 n-7, C20:1 n-9, and C20:2 n-6 were more abundant in the perirenal fat than in the muscle.

**Figure 1:**
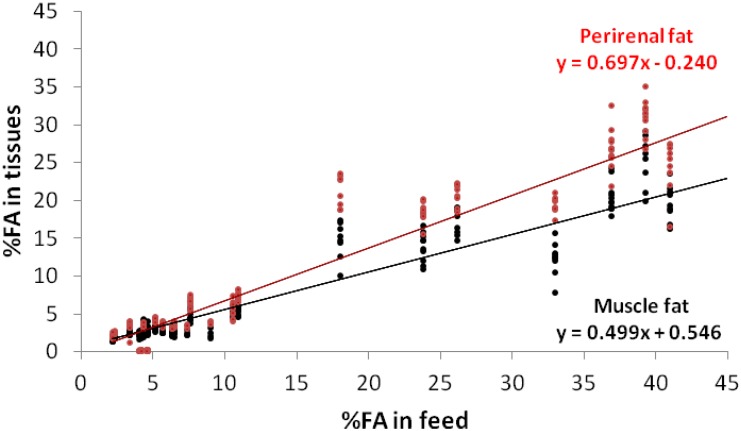
Regressions of the α-linolenic acid (C18:3 n-3) contents in the muscle fat and perirenal fat, according to their contents in the feed.

The α-linolenic acid was detected in significantly greater concentrations in the perirenal fat than in the *longissimus dorsi* muscle (Figure 1). The incorporation rate in the perirenal fat was 40% higher than in the muscle (0.7 *vs.* 0.5). Thus, the difference in the concentration between tissues only became relevant after 10% of integration. Combes [26], considering the results of 14 researches on rabbit nutrition, found a relationship (y = 0.13x + 0.92; R^2^ = 0.532) between the α-linolenic acid of rabbit meat (y) and that of the feed (x). In that paper, the range of the FA in the diets was mainly restricted to 0–15% and the response was below 6% in the muscle. Petracci *et al*. [27] reported that α-linolenic acid enrichment has the potential of providing a useful intake of n-3 PUFA to help balance the n-6/n-3 PUFA ratio in rabbit meat.

Many studies have reported that the FA profile of rabbit meat may be favourably modified through the inclusion of raw materials rich in PUFA. Ouhayoun *et al*. [28] found that soya full fat can increase the proportion of linoleic acid in the adipose tissue by 30% of the total FAs. Cobos *et al*. [29] noted that enriching the diets of rabbits with soya, sunflower oil or soya bean oil increased the proportion of unsaturated FAs compared to those obtained using conventional diets. Xiccato *et al*. [30] also observed a high level of unsaturation in rabbit fat. Oliver *et al*. [3] found a clear effect of the composition of the diet on the FA composition of rabbit fat. Szabó *et al*. [31] reported that the FA profile of the *longissimus dorsi* muscle of rabbits effectively reflected that of three types of feed: a commercial pelletted diet and two high-fat diets, one complemented with a saturated animal fat source and the other with an unsaturated fat source (full-fat soya).

**Figure 2:**
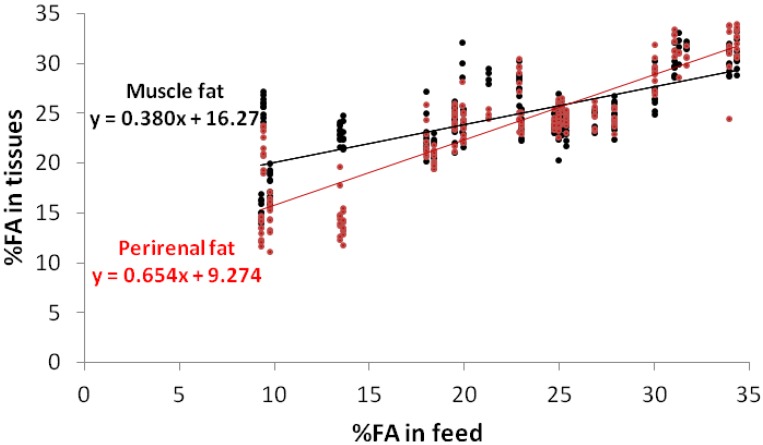
Regressions of the oleic acid (C18:1 n-9) contents in the muscle fat and perirenal fat, according to their contents in the feed.

**Figure 3:**
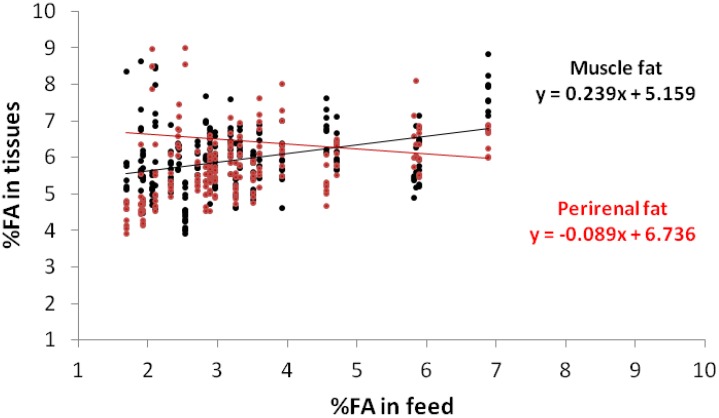
Regressions of the stearic acid (C18:0) contents in the muscle fat and perirenal fat, according to their contents in the feed.

**Figure 4:**
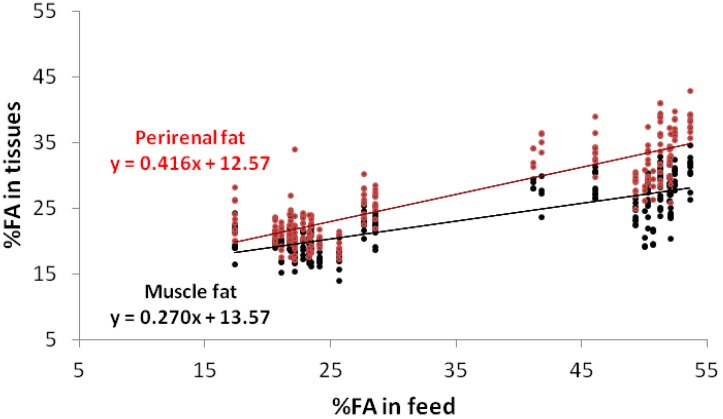
Regressions of the linoleic acid (C18:2 n-6) contents in the muscle fat and perirenal fat, according to their contents in the feed.

High levels of oleic acid in the diets of rabbits lead to an increase in this acid in the fat and a decrease in palmitic acid, which is regarded as beneficial from the human health point of view [32]. Figure 2 underlines the results of Table 3 and outlines that the higher reactivity to the diet in the perirenal fat (0.66) than in the muscle (0.39), combined with the homeostasis in the muscle, delineates a real difference in the feeding levels below the 15% level, thereafter no difference was evident. On the contrary, stearic acid showed a mild decline (−0.12), but only in the perirenal fat (Figure 3). Figure 4 shows the percentage of weight of linoleic acid in the muscle and in the perirenal rabbit fat, according to their contents in the diets with trend similar to that of α-linolenic acid.

Feeding rabbits with diets containing considerable amounts of linoleic acid leads to an increase in the amount of this acid in the perirenal fat, although only up to a certain point [27,30].

Hernández *et al*. [33] found higher percentages of linoleic and α-linolenic acid in the meat of rabbits fed a diet enriched with 3% linseed oil and 3% sunflower oil, respectively, than in the rabbits fed a diet enriched with 3% animal fat.

Gigaud and Combes [34] found a closer relationship (y = 1.58x + 21.16; R^2^ = 0.894) between the n-3 PUFA content in the meat (y) and in the feed (x).

Colin *et al*. [35] studied the influence, on meat lipids, of an increase in n-3 PUFA level in the feed with the incorporation of extruded linseed, and found a close relationship (y = 3.58x + 2.48; R^2^ = 0.938) between the n-3 PUFA content in the rabbit meat (y) and in the feed (x).

**Table 4:** Linear regression equations of the total sum of n-3 PUFA (C18:3 n-3 + C20:3 n-3 + C20:5 n-3 + C22:6 n-3) in the *longissimus dorsi* muscle (M) and in the perirenal fat (P) on percentage of the eleven fatty acids in the feed.

Independent FAs (X)		Muscle (M)			Perirenal (P)		
	R^2^	0.94			0.96		
	SE	1.86			2.06		
	Mean	7.96			9.98		
	Intercept	aM	SaM	Sign.	aP	SaP	Sign.
		−27	10.3	0.0136	−46	9.7	<.0001
	Means of X	bM	SbM	Sign.	bP	SbP	Sign.
C12:0	0.004	−10.2	8.5	0.18	−18.6	8.7	0.035
C14:0	0.43	4.5	1.5	0.0002	7.6	1.5	<.0001
C16:0	15.81	0.2	0.1	0.25	0.4	0.1	<.0001
C16:1 n-7	0.040	7.4	2.4	0.0003	7.5	2.3	0.0020
C16:1 n-9	0.24	−4.3	1.3	0.0004	−5.3	1.3	<.0001
C18:1 n-9	22.81	0.4	0.1	0.0001	0.5	0.1	<.0001
C18:2 n-6	36.43	0.3	0.1	0.047	0.4	0.1	<.0001
C18:3 n-3	13.15	0.7	0.1	<.0001	1.0	0.096	<.0001
C20:0	0.25	4.4	1.4	0.042	14.0	1.5	<.0001
C20:1 n-9	0.80	0.8	0.2	<.0001	0.3	0.2	0.21
C20:2 n-6	0.46	1.7	0.2	<.0001	2.1	0.2	<.0001

In the present work, the n-3 polyunsaturated fatty acids (n-3 PUFA) meat content followed the dietary content (Figure 1 and Table 4), but in a unusual way. In fact, considering the 11 dietary FAs significantly related to the n-3 PUFA (R^2^ = 0.94 and 0.96, for the muscle and perirenal fat, respectively), the α-linolenic acid, was the only n-3 PUFA in the diets which was incorporated at a 0.7 and 1.0 rate in the two tissues. Lauric acid showed a negative effect on the bio-accumulation of n-3 PUFA in the perirenal fat, but, conversely, palmitoleic acid (C16:1 n-7) strongly favoured an n-3 PUFA increase (7.4 and 7.5). Other FAs contributed positively, namely: miristic acid (4.5 and 7.6); oleic and linoleic acid with slight coefficients of around 0.5, but these FAs are represented massively (over the 50% of the FAs); the long-chain FAs (C20:0, C20:1 n-9, and C20:2 n-6) were also correlated to the n-3 PUFA growth in the rabbit tissues. As far as the etiology of these statistical relationships is concerned the answer may derive from common or concurrent pathways: α-linolenic and linoleic acid serve as the precursor molecules from which the set of FAs belonging to the n-3 and n-6 PUFA family can be synthesized through a series of elongation and desaturation reactions. All the reactions are catalyzed by an enzymatic system consisting of fatty acil-CoA synthetases ∆6 and ∆5 desaturases and the respective elongases. These two FA families not only share these enzymes, but they also compete for the same enzymes [36].

Reversibility of the FA profile in rabbit muscle has been studied using two dietary fat supplementations [9]. FA incorporation and reversibility in the muscle have been demonstrated and, at the end of the trial, the experimental groups only differed on the basis of the meat contents of the palmitic and linoleic acids. The restitution of the tissue FA profile, following a change in the dietary fat source, can clearly be obtained, but it depends on the magnitude of the proportional change in the FAs in the diet.

**Table 5:** Pearson correlations between the FA averages of the experimental groups.

	r			r^2^		
	Diets	Muscle	Perirenal	Diets	Muscle	Perirenal
Diets	1	0.870	0.926	1	0.756	0.856
Muscle	0.870	1	0.975	0.756	1	0.951
Perirenal	0.926	0.975	1	0.857	0.951	1

As can be seen in Table 5, considering the correlations of 3,792 diet-muscle-perirenal fat triplets concerning 16 available FAs, the profiles of the two tissues appeared to be very similar to each other (r^2^ = 0.951), but the feeding varieties had a greater impact on the perirenal fat (r^2^ = 0.857) and, to a lesser extent, on the muscle (r^2^ = 0.756) FA layout; essentially, the overall difference between the tissues in the reflection of the characteristics of the diet, amounted to 10%.

## 4. Conclusions

The FA profile was clearly influenced by diet composition and it was possible to linearly characterize the incorporation of certain FAs. Our studies on rabbit meat indicate that the dietary inclusion of various raw materials could be considered as a way of achieving the enrichment of both linoleic and α-linolenic acid in the rabbit meat. Further research is needed to identify the optimum conditions necessary to obtain the required dietary results at lower costs. A proposal for the prediction of n-3 PUFA from dietary α-linolenic acid and a panel of another ten FAs has been made.
